# Potential therapeutic effects of baicalin and baicalein

**DOI:** 10.22038/AJP.2023.22307

**Published:** 2024

**Authors:** Kamyar Sabry, Zahra Jamshidi, Seyed Ahmad Emami, Amirhossein Sahebka

**Affiliations:** 1 *Department of Pharmacognosy, School of Pharmacy, Mashhad University of Medical Sciences, Mashhad, Iran*; 2 *Department of Medicinal Chemistry, School of Pharmacy, Mashhad University of Medical Sciences, Mashhad, Iran*; 3 *Department of Traditional Pharmacy, School of Pharmacy, Mashhad University of Medical Sciences, Mashhad, Iran*; 4 *Applied Biomedical Research Center, Mashhad University of Medical Sciences, Mashhad, Iran*; 5 *Biotechnology Research Center, Pharmaceutical Technology Institute, Mashhad University of Medical Sciences, Mashhad, Iran *; 6 *Department of Biotechnology, School of Pharmacy, Mashhad University of Medical Sciences, Mashhad, Iran*

**Keywords:** Baicalin, Baicalein, Hepatoprotective, Cancer, Metabolic syndrome, Neuroprotective

## Abstract

**Objective:**

Baicalin and baicalein are natural flavonoids reported for the first time from *Scutellaria baicalensis* Georgi. Recently, attention has been paid to these valuable flavonoids due to their promising effects. This paper aims to have a comprehensive review of their pharmacological effects.

**Materials and Methods:**

An extensive search through scientific databases including Scopus, PubMed, and ISI Web of Science was established.

**Results:**

According to literature, these compounds have been mainly effective in the treatment of neurological and neurodegenerative diseases, hepatic and cardiovascular disorders, metabolic syndrome, and cancers through anti-inflammatory and antioxidant pathways. Induction of apoptosis and autophagy, and inhibition of migration and metastasis are the main mechanisms for their cytotoxic and antitumor activities. Decreasing inflammation, reducing oxidative stress, regulating the metabolism of lipids, and decreasing fibrosis, apoptosis, and steatosis are their main hepatoprotective mechanisms. Inhibiting the development of cardiac fibrosis and reducing inflammation, oxidative stress, and apoptosis are also the mechanisms suggested for cardioprotective activities. Decreasing the accumulation of inflammatory mediators and improving cognitive function and depressive-like behaviours are the main mechanisms for neurological and neurodegenerative activities.

**Conclusion:**

The findings suggest the therapeutic potential of baicalin and baicalein. However, complementary research in different *in vitro* and *in vivo* models to investigate their mechanisms of action as well as clinical trials to evaluate their efficacy and safety are suggested.

## Introduction

**Figure 1:**
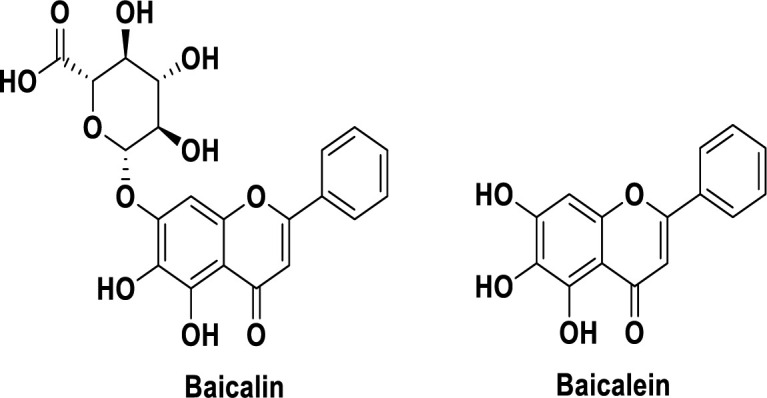
The chemical structure of baicalin and baicalein.

Medicinal plants and their phytochemicals have always been an inspiring source of pharmaceutical lead compounds and drug candidates against various human diseases (Newman and Cragg, 2020). Baicalin and baicalein are natural products belonging to the flavonoid class of specialized metabolites (flavone subfamily). Baicalin is 5, 6-dihydroxy-7-O-glucuronide flavone, and baicalein is its aglycone (Figure 1). These compounds are the main flavonoids in the roots of *Scutellaria baicalensis* Georgi. Phytochemical studies revealed these compounds in other *Scutellaria* species such as *S. lateriflora* L. (Tuan et al., 2018), *S. viscidula* Bunge (Wang et al., 2008), and *S. rivularis* Benth. (Lin and Shieh, 1996) as well as from other genera including *Oroxylum indicum* (L.) Benth. ex Kurz (Bignoniaceae) or Indian trumpet flower. Flavonoids are one of the main active constituents of these valuable medicinal herbs (Maleki et al., 2022). Phytochemical investigation of other *Scutellaria* spp. and evaluation of their flavonoid content including baicalein and baicalin might reveal their potential as a substitute for *S. baicalensis*.

*Scutellaria* spp. has a long reputation for use in different systems of traditional medicine (Maleki et al., 2022). *Scutellaria baicalensis* has been used for thousands of years in traditional Chinese medicine (TCM) to treat inflammation, fever, jaundice, and hypertension (Maleki et al., 2022). In recent years, much attention has been paid to investigating the therapeutic potential of *Scutellaria* spp. and their active metabolites particularly baicalin and baicalein due to their diverse pharmacological activities like antioxidant, anti-tumor, anti-viral, anti-HIV, anti-inflammatory, and anti-proliferative effects (Shen et al., 2021). However, no comprehensive study has reviewed the beneficial activities of these valuable compounds. In this review article, we summarized several studies according to PubMed and Scopus databases to evaluate the therapeutic potential of baicalin and its aglycone baicalein on different disorders.

## Materials and Methods

**Search strategy**

Web of Science (ISI), PubMed, and Scopus were searched to collect all studies reporting the pharmacological activities of baicalin and baicalein from 2015 to 2022. Keywords used for the search were all relevant pharmacological effects in combination with "baicalin" or "baicalein".

## Results

**Pharmacology**

**Liver problems**

About two million people die from the liver disease every year. This number equates to 3.5 percent of all deaths globally (Asrani et al., 2019). Recent studies show that baicalin and baicalein have therapeutic potential to treat liver problems. In the following paragraphs, we review their effect on the treatment of different liver disorders.

**Non-alcoholic fatty liver disease (NAFLD)**

One of the most common liver diseases in the world is NAFLD. Hepatic fat accumulation is the most important cause of NAFLD. There are many pathological factors for liver fat accumulation and NAFLD. Oxidative stress is an important factor in NAFLD pathogenesis. Reducing oxidative stress and protecting the mitochondria to inhibit apoptosis are proposed as hepatoprotective mechanisms of baicalin in NAFLD (Gao et al., 2022). In this study, baicalin could inhibit apoptosis and lactate dehydrogenase (LDH) release. Baicalin could reduce the levels of ROS and fatty acid-induced MDA, and increase superoxide dismutase (SOD) and glutathione amounts compared to the control. Moreover, baicalin could partially restore mitochondrial morphology and increase ATP5A expression and mitochondrial membrane potential (Gao et al., 2022). As above mentioned, NAFLD is caused by inflammation after fat accumulation. During inflammation, the most abundant immune cells engaged in improving the injury are macrophages. Studies show that baicalin could polarize macrophages into anti-inflammatory M2c subtype macrophages with higher expression level of MERTK. In a mice model, the potential and efficacy of baicalin-induced MERTK^+^/hi M2c macrophages were evaluated as a cell-based therapy for NAFLD (Junior et al., 2021). They found that an injection of MERTK^+^/hi M2c macrophages to NAFLD mice could decrease the circulating CD4^+^CD25- and CD8^+^CD25- T cells, increase serum HDL levels in the liver, and lower the total NAFLD pathological score. In addition, the injection of baicalin could downregulate profibrotic COL1A1, FN, TNF-α, and PPAR-γ expression (the regulator of lipid metabolism) (Junior et al., 2021). Baicalein has also therapeutic effects against NAFLD. Administration of baicalein in mice with NAFLD has shown inhibitory effects against NAFLD by modulating the structure of the gut microbiota. After baicalein treatment, a remodelling in the overall structure of the gut microbiota was observed and the upregulated expressions of numerous genes in hepatocytes such as Pla2g12a, Apoa4, Slc27a4, Elovl7, Fabp4, Hilpda, Gpld1, Vldlr, and Apom were restored. In addition, baicalein could regulate the metabolism of lipids like 2-oxocarboxylic acid, α-linolenic acid, and pantothenate, and the secretion of bile (Li et al., 2022). Activating AMPK is one of the crucial mechanisms by which baicalein exerts its effects on NAFLD. In a study by Sun et al., the hepatic fat-lowering activity of baicalein was investigated. Baicalein could alleviate fat accumulation in the liver by suppressing SREBP1 cleavage and activating AMPK. As a result, baicalein could inhibit the SREBP1 transcriptional activity and the hepatic fat synthesis *in vitro* and *in vivo*. Besides, decreasing total cholesterol (TC) and low-density lipoprotein cholesterol (LDLC), increasing high-density lipoprotein cholesterol (HDLC), and regulating the antioxidant activity were other outcomes (Sun et al., 2020). Attenuating the impairment of hepatic lysosomal acidification via maintaining V-ATPase assembly is another mechanism suggested for the hepatoprotective activity of baicalein against NAFLD (Zhu et al., 2020). Moreover, baicalin has exhibited potential anti-NAFLD in combination with other drugs. For example, a combination of baicalin, berberine, and puerarin showed promising effects against NAFLD in a rat model. The study indicated that interleukin-6 (IL-6) and TNF-α levels in serum were modified in the baicalin group. Additionally, the expression levels of PPAR-γ/IR protein and mRNA were decreased by the combination (Zhao et al., 2016). Similarly, a recent study revealed the potential of a baicalein-acarbose combination to inhibit *de novo* lipogenesis and the expression of fatty acid synthase (Xing et al., 2021). 

**Non-alcoholic steatohepatitis (NASH)**

Several studies have confirmed that baicalin and baicalein have therapeutic effects for treating NASH. Different mechanisms have been proposed for their activity. In a mouse model, baicalin remarkably decreased inflammation, hepatic steatosis, apoptosis, fibrosis caused by Methionine–choline-deficient (MCD) diet. Baicalin treatment notably could reduce hepatic lipid accumulation induced by MCD diet partly by adjusting the expression of FASN, PPARα, SREBP-1c, and CPT1α. Baicalin treatment enormously inhibited methionine deficiency and hepatic inflammation associated with a reduction in IL-1β, TNF-α, and macrophage influx, MCP-1 production, and NF-κB activation. Additionally, baicalin could block liver fibrosis mediated by inhibition of transforming growth factor beta (TGF-β1), Col1A1, and α-SMA. Additionally, baicalin could remarkably decrease caspase-3 protein expression and inhibit hepatocyte apoptosis in methionine-choline deficient (MCD) mice (Zhang et al., 2018). According to the literature, administration of baicalin could decrease fibrosis, proinflammatory cytokines, oxidative stress, profibrotic gene expression, and cell death. In addition, it could improve the mitochondrial electron transport chain functionality and NRF2 activation by nuclear translocation and induce its target genes GCLM and HO-1, leading to increased antioxidant defence capacity (Shen et al., 2017). In addition, baicalin could control the NRF2/HO-1/NRLP3 pathway to reduce hepatic cell pyroptosis both *in vivo* and *in vitro* (Shi et al., 2022).

**Viral hepatitis**

About 80,000 patients per 240 million patients who are infected by the hepatitis B virus die every year (Ropero Álvarez et al., 2017). Baicalin has a promising antihepatitis effect. Studies have revealed that baicalin can increase the survival of duck embryonic hepatocytes infected with duck hepatitis A virus by inhibiting virus replication and suppressing hepatitis B virus RNA transcription. Additionally, baicalin could downregulate HNF-1 and HNF-4 expression levels (Huang et al., 2017; Chen et al., 2019; Xia et al., 2020). Another study showed that the administration of baicalin (75 g/ml) could significantly reduce inflammation and the replication of hepatitis B virus in HuH7 and HepG2 cells by downregulating the NF-κB signalling pathway (Pollicino et al., 2018). Hepatocyte nuclear factors (HNFs) are approved to be necessary for hepatitis B virus replication. In the hepatitis B virus core promoter, HNF4 binds to HNF4BE(s) and subsequently promotes the transcription of viral hepatitis B pregenomic RNA (pgRNA). It has also been shown that the combination of baicalin and entecavir was more effective than either agent alone in suppressing the virus, stimulating a natural immune response, and reducing viral hepatitis B-related liver inflammation. The antiviral hepatitis B effect of baicalin might be via downregulating HNF4 and HNF1 leading to decreased RNAs of the hepatitis B virus (Huang et al., 2017).

**Hepatocellular carcinoma** **(HCC)**

HCC is among the most malignant types of cancer. In recent years, the occurrence of HCC has increased (Robinson et al., 2019). Baicalin as a hepatoprotective agent is suggested to have the potential to treat HCC by inducing cell death and autophagy in tumour cells. In *in vitro* studies, baicalin could reduce HCC cell survival and proliferation by decreasing the expression of certain proteins included in cell growth (cyclin-dependent kinase 2, cyclin A, a and cyclin D1), as well as triggering cell apoptosis (Yu et al., 2015; Yu et al., 2016). In addition, baicalein is believed to block the ERK signalling pathway which is responsible for the reduction in matrix metalloproteinas-2 (MMP-2), matrix metalloproteinase-9 (MMP-9), and the expression of u-PA ( urokinase-type plasminogen activator) (Chen et al., 2013).

**Liver ischemia-reperfusion** **(IR)**

IR injury, referring to damage caused by reducing blood flow and decreasing oxygen delivery to cells, is aggravated by ROS and other inflammatory mediators (Wang et al., 2014). Recent studies have revealed that baicalin, due to its antioxidant and anti-inflammatory properties, might be a potential treatment for liver injury caused by oxidative stress and inflammation. In a study, baicalin pretreatment could decrease the expression of caspase 3, cleaved caspase 3, and Bax while upregulating the Bcl-2 expression. Furthermore, baicalin pretreatment could reduce CHOP and BiP and enhance beclin-1 and LC3 notably. In addition, baicalin could attenuate serum ALT activity, IL-6 levels, and TNF-α in alcoholic fatty liver disease (Kim and Lee, 2012; Liu et al., 2018). It has also been shown that baicalein can suppress the NF-B signalling pathway (Liu et al., 2015). Thus, it can be concluded that baicalin may protect liver IR by reducing oxidative stress and inflammation.

**Cholestatic liver disease**

Cholestasis is a disorder of the flow of bile that can be caused by a variety of factors. It is characterized by the accumulation of toxic bile acids in the liver, which can damage hepatocytes and the bile ducts. Cholestasis can progress to liver fibrosis or liver failure if left untreated (Gossard and Talwalkar, 2014). Baicalin has exhibited protective effects in a cholestatic mouse model by reducing inflammation, oxidative stress, and fibrosis. Baicalin could enhance antioxidant defence capacity via activating NRF2 and its target genes heme oxygenase-1 (HO-1) and glutamate cysteine ligase (GCLM). Besides, baicalin was able to alleviate the activation of stellate cells, which is critical for the initiation of fibrosis (Shen et al., 2017). 

**Infections**

**Viral infections**

Viral infections are one of the major health problems of all time. The recent pandemic has affirmed that natural products have critical roles in treating viral infectious conditions. In the last two years, SARS-CoV-2 infection has become a serious health concern worldwide with more than 6 million deaths in the world. During this pandemic, the shortage of antiviral drugs has spurred greater use of herbal medicines in some parts of the world, like Africa (Rackimuthu et al., 2021). In an *in vitro* study, four important constituents from *S. baicalensis* consisting of wogonin, baicalein, baicalin, and wogonoside could inhibit the activity of SARS-CoV-2 3CLpro. Baicalein displayed the greatest activity against SARS-CoV-2 3CLpro (IC_50_ of 0.39 µM) (Liu et al., 2021). One of the mechanisms by which baicalin and baicalein may exert their antiviral effect is by inhibiting SARS-CoV-2 RdRp ( RNA-dependent RNA polymerase) (Zandi et al., 2021). 

Dengue is mosquito-borne that caused by the dengue virus (DENV) (Gibbons and Vaughn, 2002). Zandi et al. have shown that baicalein had significant inhibitory activity on DENV-2 replication in Vero cells in all stages of the DENV-2 replication cycle including intracellular virus replication and adsorption stage (Zandi et al., 2012; Moghaddam et al., 2014). Moreover, baicalein is reported to have fundamental antiviral activity against the diverse stages of *in vitro* Japanese Encephalitis Virus with IC_50_=14.28 μg/mL and IC_50_=3.44 μg/mL. Baicalin has also been shown to be effective against viral infections. In a study, baicalin displayed inhibitory activity against EV71 infection by downregulating the expression of EV71/3D mRNA polymerase at the early stages of the infection (Yang et al., 2015). Moreover, it could reduce the expressions of caspase-3 and FasL (Johari et al., 2012; Yang et al., 2015; Yang et al., 2021). Modulation of the expression levels of interleukin, SOCS1/3 (suppressor of cytokine signaling), IFN (interferon), PKR (protein kinase R), Mx1 protein (human GTP-binding protein), regulation of TLRs (toll-like receptor), JAK/STAT (janus kinase/ signal transducer and activator of transcription), and NF-κB (nuclear factor kappa β) pathways, and inhibition of the virus infection-induced cell apoptosis are some proposed mechanisms for antiviral activity of baicalin (Li et al., 2021).

**Fungal infections**

Fungal infections are important infectious problems threatening human life. Candidiasis is a prominent fungal infection caused by *Candida* spp., with a very challenging treatment (Mokaddas et al., 2007; Pfaller and Diekema, 2010; Serpa et al., 2012). There are a wide variety of medications such as fluconazole that are considered first-line therapy for fungal problems. However, the efficacy of several natural products including baicalin and baicalein has been evaluated to discover new promising antifungal agents (Table 1). For instance, a combination of fluconazole and baicalein could increase the effectiveness of flocculent extracellular material production with a biofilm-like structure (Hajjeh et al., 2004). Moreover, baicalein has exerted significant inhibitory activity against *Candida albicans* by disrupting glycolysis via targeting Eno1 (Li et al., 2022). Exposure to baicalein at MIC_50_ values exhibited anti-candidal activity against *C. albicans* and other *Candida* species (Serpa et al., 2012; Shirley et al., 2017; Li et al., 2022). Utilization of non-cytotoxic concentrations of baicalein showed a significant fungal inhibitory activity and reduced fungal load. Furthermore, mRNA and protein levels of proinflammatory factors such as TNF-α, IL-1β, and IL-6, were also suppressed after baicalein treatment (Zhu et al., 2021). Similarly, baicalein has shown protective activity against *Aspergillus fumigatus* keratitis (Zhu et al., 2021). 

**Bacterial infections**

Studies show that baicalin and baicalein have antibacterial activities against different pathological bacteria such as Avian pathogenic *Escherichia coli* (Peng et al., 2019), *Escherichia coli*, *Staphylococcus aureus* (Gao et al., 2017; Zhang et al., 2020; Miao et al., 2021), *Helicobacter pylori*, and *Chlamydia trachomatis* (Hao et al., 2012) (Table 2). For instance, baicalin can inhibit biofilm formation, decrease the expression of associated genes, and regulate the levels of IL-1β, IL-4, and TNF-α via the suppression of NF-κB pathways (Peng et al., 2019). 

**Cancer**

**Figure 2:**
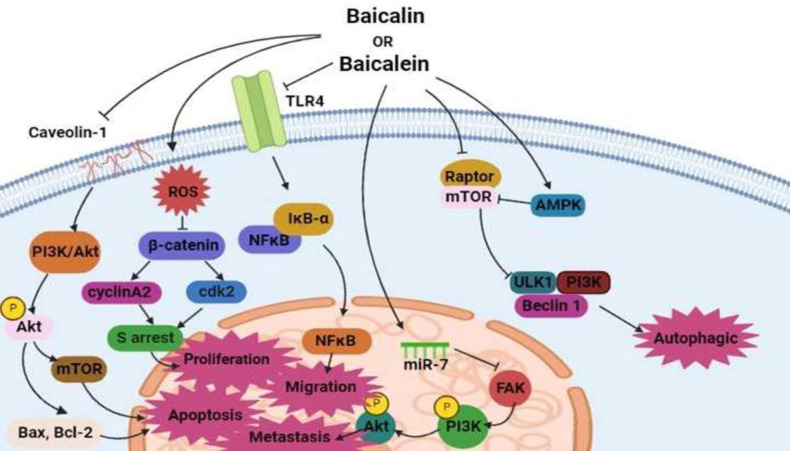
Baicalein/baicalin might be autophagy inducers by activating the AMPK/ULK1 pathway and negatively regulating the mTOR complex, and apoptosis inducers by interrupting the caveolin-1/Akt/mTOR. They might also inhibit the proliferation process by suppressing β-catenin signaling pathway, and reduction of migration and invasion by suppressing TLR4/NF-κB pathway, and metastasis by mediating miR-7/FAK/AKT pathway.

Cancer is an important cause of death in the world with about 10 million fatalities in 2020, accounting for almost one in every six deaths (Singh et al., 2021). Because of their low toxicity and high efficiency, dietary flavonoids are gaining popularity as potential anticancer agents (Moore et al., 2016). It has been revealed that baicalin and baicalein have cytotoxic and anti-tumour activities through different signalling pathways (Figure 2).

**Table 1:** Antifungal activities of baicalein

Fungi	Compd.	Mechanism(s)	Ref.
*Candida albicans*	Baicalein	↓ Cell viability and cell growth	(Serpa et al., 2012)
	Baicalein	Disrupting glycolysis through targeting Eno1	(Li et al., 2022)
	Baicalein	↓ Total growth, metabolic activity, biofilm formation, and cell surface hydrophobicity	(Shirley et al., 2017)
*Candida tropicalis*	Baicalein	↓ Cell viability and cell growth	(Serpa et al., 2012)
*Candida parapsilosis*	Baicalein	↓ Cell viability and cell growth	(Serpa et al., 2012)
*Aspergillus fumigatus*	Baicalein	↓ Fungal load, TNF-α and IL-6 Inhibited neutrophil infiltration, and the expression of TSLP and TSLPR	(Zhu et al., 2021)

**Table 2:** Antibacterial activities of baicalin or baicalein

Bacteria	Compd.	Mechanism(s)	Ref.
***Escherichia coli***	Baicalin	↓ AI-2 secretion, inhibiting the expression of proinflammatory cytokines and NF-κB activation	(Peng et al., 2019)
	Baicalin	↓ TNF-α, IL-1β, iNOS, IL-10, and IL-4	(Miao et al., 2021)
***Staphylococcus aureus***	Baicalin	↓* Staphylococcus aureus* counts	(Gao et al., 2017)
	Baicalin	Inhibiting *Staphylococcus aureus* growth	(Zhang et al., 2020)
	Baicalin	↓ TNF-α and IL-1β, iNOS, IL-10, and IL-4	(Miao et al., 2021)
***Staphylococcus epidermidis***	Baicalein	Antibacterial activity	(Palierse et al., 2021)
***Helicobacter pylori***	Baicalin	Inhibitory activity via targeting sulfhydryl groups around the active site of *Helicobacter pylori*	(Yu et al., 2015)
***Mycobacterium tuberculosis***	Baicalin	Inhibiting the PI3K/Akt/NF-κB pathway, and phosphorylation of Akt	(Zhang et al., 2017)
***Chlamydia*** ***trachomatis***	Baicalin	↓ NO, PGE, and NF-κB p65 mRNA Downregulating the expression of TLR4 mRN and TLR2 mRNA	(Hao et al., 2012)

The main mechanisms of action are induction of autophagy and apoptosis, and inhibition of metastasis and migration. For example, baicalin has exhibited cytotoxic effects on T24 bladder cancer cells by blockage of Akt phosphorylation, activation of the mitochondria-dependent caspase pathway, and induction of apoptosis (Li et al., 2013). It could also significantly inhibit migration and promote mitochondria-mediated apoptosis through the TLR4/NF-κB signalling pathway in HCT-116 and CT26 cells (Song et al., 2022). In addition, baicalin could significantly reduce the proliferation and invasion of cells by increasing ROS to inhibit β-catenin signalling pathways in osteosarcoma cell lines (Pang et al., 2022).

Baicalein has also exhibited significant cytotoxic activity *in vitro*. A study has reported that baicalein could induce autophagy in MDA-MB-231 cells by downregulation of mTORC1 complex components and AMPK/ULK1 activation (Aryal et al., 2014). It could also display a strong cytotoxic effect on PC-3 and DU145 cells via interrupting the caveolin-1/Akt/mTOR pathway (Guo et al., 2015). In gastric cancer cell lines, baicalein appeared to regulate focal adhesion kinase to affect the proliferation of the cells through PI3K/AKT signalling pathway (Qiao et al., 2022). 

Interestingly, the combination of baicalein and baicalin has cytotoxic effects on several cancerous cells. As an example, the combination could induce apoptosis in breast cancer cells via activating caspase-3 and -9, decreasing Bcl-2 levels, and increasing the p53 and bax levels (Zhou et al., 2009). In another study on human colorectal adenocarcinoma cells (HCT116), baicalin and baicalein could activate caspase-3 and -9, leading to cell cycle arrest at the S phase and apoptosis (Guo et al., 2015). Besides, a combination of baicalin and doxorubicin could elevate the chemosensitivity of MCF-7 and MDA-MB-231 breast cancer cells (Lin et al., 2021).

**Cardiovascular diseases (CVD)**

CVDs are one of the greatest causes of global death, leading to the deaths of 17.9 million people each year (more than 75 percent of them are from low and middle-income countries). Cerebrovascular disease, coronary heart disease, rheumatic heart disease, and other heart and blood vessel illnesses are all classified as CVDs. More than four-fifths of cardiovascular deaths are due to stroke and heart attack. About one-third of these deaths occur before the age of 70 (Gaziano, 2005). In the following paragraphs, we discuss the beneficial effects of baicalin and baicalein on CVDs.

**Heart failure (HF)**

Heart failure is a clinical condition caused by heart problems that affect ventricular filling, or the ejection of blood into the systemic circulation. HF is a common condition with a high morbidity and death rate across the world. It is projected to affect 26 million individuals globally and adds to rising healthcare expenses (Savarese and Lund, 2017). Cardiac fibrosis is a risk factor for HF in both ischemic and non-ischemic cardiomyopathies. Cardiac fibrosis can be characterized by a rise in the number of interstitial fibroblasts and the excessive production and deposition of cardiac extracellular matrix proteins (Fan et al., 2012; Prabhu and Frangogiannis, 2016). Baicalin may help to reduce cardiac fibrosis by reducing extracellular matrix formation and the expression of genes associated with fibrosis (Zhang et al., 2017). In another research, it has been shown that baicalin may help to reduce the proliferation of fibroblasts and the accumulation of extracellular matrix, which in turn, may help to inhibit the development of cardiac fibrosis via AMPK/TGF-b/Smads signaling pathway (Xiao et al., 2018). In addition, by stimulating AMPK, baicalin can block the TGF-/Smads signaling pathway, which is a critical mechanism in the development of cardiac fibrosis (Xiao et al., 2018).

**Myocardial ischemia reperfusion injury (MIRI)**

MIRI is described as tissue damage in the ischemic myocardium following the occurrence of acute coronary artery blockage. MIRI is the main reason for death after an acute myocardial infarction (Liu et al., 2019; Li et al., 2021). Baicalin can protect cardiomyocytes from hypoxia/reoxygenation injury by elevating the SOD activity and anti-inflammatory responses through reducing TNF-α, enhancing IL-10 levels, decreasing IL-6, and inhibiting the translocation of NF-κB to the nucleus. Baicalin has dose-dependent protection against heart tissue injury caused by myocardial infarction and cardiac IR by reducing oxidative stress, inflammation, and apoptosis. In an animal model, baicalin could protect rats against IRI by its antioxidant activity. Baicalin-treated groups could improve left ventricular function, decrease lactate dehydrogenase and creatine kinase (CK) release in the coronary effluent, and elevate the activity of MDA and SOD (Lin et al., 2010; Kong et al., 2014). In an investigation, the administration of baicalin intragastrically could reduce I/R-induced myocardial damage dose-dependently, which in turn promoted PI3K/Akt pathway and suppressed NF-B signaling (Luan et al., 2019). In addition, baicalin could protect cardiac microvascular endothelial cells *in vivo* via stimulating the production of NO through the PI3K-AKT-eNOS signaling pathway and reducing necroptosis through suppressing the protein expression of RIP3, RIP1, and p-MLKL (Bai et al., 2019).

**Immune system problems**

A response of the immune system, characterized as a protective mechanism, is defined as inflammation. However, chronic inflammation that is caused by long-term pressures such as infection, physiological stress, or malnutrition can result in tissue remodeling (Fukuda and Sata, 2021). Chronic inflammatory diseases including diabetes mellitus, ischemic heart disease, stroke, chronic kidney disease, cancer, NAFLD, and neurodegenerative diseases have been identified as the main reason of mortality in the world (Furman et al., 2019). In various pieces of research, anti-inflammatory and immunomodulatory effects have been reported for baicalin and baicalein in different diseases, some of which are mentioned below.

**Inflammatory bowel disease (IBD)**

IBD is a class of chronic gastrointestinal inflammation including Crohn's disease and ulcerative colitis. According to reports, one-third of patients with ulcerative colitis and two-thirds of patients suffering from Crohn's disease will need surgery at some point (Ali et al., 2012; Pineton de Chambrun et al., 2016). It is believed that the occurrence of IBD is greater in developed countries. According to 2017 estimates, over six million instances of IBD were reported worldwide (Alatab et al., 2020). IBD is characterized by an immunological imbalance of the intestinal mucosa in response to self-antigens, leading to chronic inflammatory situations. Considering that, an optimal effect on immune cells is essential in the therapy for this problem (De Mattos et al., 2015). 

Studies show that baicalin and baicalein may be effective against IBD by suppressing oxidative stress and inflammation, and regulating the immune system. Baicalin and baicalein have exhibited effects on many immunologic pathways. For instance, baicalein can improve the symptoms of ulcerative colitis by lowering the expression of pregnane X receptor (PXR), inducible nitric oxide synthase (iNOS), cyclooxygenase-2 (COX-2), and caudal-type homeobox 2 (Cdx2), as well as the NF-κβ and STAT3 pathways. It could increase the subsequent degradation of IκBα and attenuate the activity and phosphorylation of IKKβ. In addition, it could inhibit the phosphorylation and nuclear translocation of p65, decrease the DNA binding activity of NF-kβ, and additionally block the expression of cyclin D1 (Zhong et al., 2019). Moreover, for baicalin, similar results were observed. Administration of baicalin (30-90 mg/kg) could decrease the levels of prostaglandin E2 (PEG2), myeloperoxidase (MPO), IL-1β, TNF-α, and the apoptosis-related genes including Bcl-2 and caspase-9 (Shen et al., 2019).

Studies show that baicalin and baicalein could decrease serum concentrations of the inflammatory markers of SOD, MDA, plasma glutathione peroxidase (GSH-P X), IL-1, IL-6, and IL-17. In addition, a combination of baicalin and baicalein could provide better results in treating ulcerative colitis (Liang et al., 2019). Macrophage migration inhibitory factor (MIF), as a cytokine, has an essential role in IBD development (Gordon-Weeks et al., 2015). In a rat model of ulcerative colitis, baicalin could decrease the expression of MIF, the amount of macrophage related cytokines and the number of macrophages, macrophage inflammatory protein-3α, and macrophage chemotactic factor-1 (Dai et al., 2012).

**Intracerebral hemorrhage (ICH)**

ICH is the main reason of 10–15% of all strokes and is linked to considerable mortality and morbidity (Zhou et al., 2014). Baicalin may help to reduce inflammation and secondary brain damage following ICH. Based on a study on male rats, baicalin may help to reduce brain damage and improve outcomes following ICH stroke (Zhou et al., 2014). In this study, baicalin could significantly alleviate ICH and reduce the release of IL-6 and IL-1 following ICH. Additionally, baicalin was found to inhibit MMP-9 activity (an enzyme involved in the tissue breakdown) (Zhou et al., 2014). In addition, baicalein could suppress oxidative stress and prevent brain damage following ICH by stimulating the Nrf2/ARE pathway via the miR-106a-5p/PHLPP2 axis (Tang et al., 2022). Moreover, it could reduce ICH-induced brain damage by preventing ROS and the NLR family pyrin domain-containing 3 (NLRP3) inflammasome (Chen et al., 2022).

**Rheumatoid arthritis (RA)**

RA, with a prevalence of 1% worldwide and as a chronic inflammatory autoimmune condition, generally affects the smaller joints (Bullock et al., 2018). JAK/STAT3 signalling pathways possibly have roles in collagen-induced arthritis pain models in mice. Baicalin remarkably could decrease disease activity in a RA mouse model by supressing relevant proinflammatory cytokines such as IL-1b, IL-6, MMP-2, MMP-9, TNF-α, iNOS, and COX-2). Moreover, baicalin could induce cell apoptosis in synovial fluid monocytes and noticeably decrease JAK1/STAT3 in the synovium of arthritis. Another study displayed that baicalin could improve the SR-BI (scavenger receptor class B type 1) expression and induce cholesterol efflux in macrophages via a PPAR-γ/LXRα/SR-BI signalling (Yu et al., 2016; Wang et al., 2018). In another study, baicalin could significantly block protein expression and phosphorylation of NF-kB p65 in human-derived synoviocytes and synovial tissue (Wang et al., 2014). 

**Metabolic disorders**

Metabolic syndrome, a collection of metabolic abnormalities, is considered a serious public health problem globally. The beneficial activities of baicalin and baicalein on metabolic disorders have been investigated in several cellular and animal studies. Baicalin and baicalein may be favorable candidates for preventing and treating metabolic disorders by upregulation and activation of PPAR-γ and AMPK as important pathways in the glucose hemostasis and lipid metabolism and by exerting anti-inflammatory responses (Baradaran Rahimi et al., 2021). In the following paragraphs, the effects of baicalin and baicalein for the treatment of different components of metabolic disorders are discussed.

**Diabetes mellitus**

Diabetes mellitus is a metabolic disorder accompanied by a raise in the levels of blood glucose (Kesika et al., 2019). The beneficial antidiabetic activities of baicalin and baicalein have been investigated in several preclinical studies. Decreasing resistance to insulin secretion from β-cells, reducing hyperglycemia (Fu et al., 2014; Kwak et al., 2017; Li et al., 2017), inhibiting the carbohydrates hydrolysable enzymes (including α-glucosidase and α-amylase) in the gastrointestinal tract (Yang et al., 2015; Li et al., 2018) and increasing GLUT4 expressions and translocation (Yu et al., 2016; Wang et al., 2017; Yang et al., 2019) are some of the proposed mechanisms for anti-diabetic activities of baicalin and baicalein (Kesika et al., 2019). Besides, these compounds have exhibited beneficial effects on the complications of diabetes such as retinopathy (Dai et al., 2019), nephropathy and neuropathy, and macrovascular diseases, containing coronary heart disease, high blood pressure, atherosclerosis, and cerebrovascular disorders through decreasing oxidative stress (Ma et al., 2015; Zhang et al., 2018).

**Hyperlipidemia**

While hyperlipidemia is characterized by raised levels of LDL, TG, and cholesterol and decreased levels of HDL macroscopically (Mollazadeh et al., 2019), various changes in cell membrane features are accompanied at the microscopic level. For instance, hyperlipidemia promotes the peroxidation of fatty acids and cell membrane cholesterol content while reducing the ATPase activity and membrane fluidity in erythrocytes. This makes erythrocytes less flexible and increases aggregation. Studies show that baicalin could decrease lipid peroxidation and activate AMPK (Kahn et al., 2005). Baicalein has also shown similar activities. For instance, oral administration of baicalein (400 mg/kg/day) in high-fat diet mice could remarkably alleviate body weight as well as serum glucose, LDL, ALT (alanine transaminase), TG (triglyceride), AST (aspartate transaminase), and monocyte chemoattractant protein-1 (MCP-1) levels. It also could decrease SREBP-1, PPAR-γ, FAS (fatty Acid Synthase), AP2 (adiponectin), and Nrf2 mRNA levels, as well as p-JNK1/2/3 and p-ERK1/2 levels (Pu et al., 2012). Interestingly, baicalin and baicalein could reduce hyperlipidemia both *in vitro* and *in vivo*. For example, oral administration of baicalin (100, 200, and 400 mg/kg/d) in high-fat diet mice could diminish the body and liver weight, TC, serum levels of LDL, TG, AST, ALT, and liver steatosis, while it could increase the hepatic phosphorylation of acetyl-CoA carboxylase (ACC), AMP-activated protein kinase (AMPKα), and CaMKKβ (Xi et al., 2015). 

**Obesity**

Excessive lipid accumulation is defined as obesity which is one of the most important public health concerns over the last decades. Obesity is a known risk factor for many health conditions, including hypertension, hyperlipidemia, diabetes mellitus, stroke, sleep apnea, coronary heart disease, osteoarthritis, and some cancers (DiBaise and Foxx-Orenstein, 2013). Baicalin and baicalein have shown anti-lipogenic and anti-obesity activities through different signaling pathways. Fat cell formation, referred to as adipogenesis, is an important factor to control body fat mass (Ntambi and Young-Cheul, 2000). Studies show that baicalin has anti-adipogenesis activity by preventing the differentiation of 3 T3-L1 pre-adipocytes into adipocytes, downregulating the expression of C/EBPα and PPAR-γ, reducing the gene and protein expression of FABP-4, lipoprotein lipase, adipsin, perilipin, and ADIPOQ, enhancing the protein expression of C/EBP homologous, C/EBPγ, and KLF-2, and mitigating the pro-adipogenic KLF-15 expression levels in 3 T3-L1 cells (Rosen and MacDougald, 2006; Lee et al., 2009). Similarly, baicalein could reduce adipogenesis. It has been shown that baicalein could decrease fat accumulation via inhibiting the differentiation of 3 T3-L1 pre-adipocytes into adipocytes, reducing mRNA expression levels of APO D, phospholipid scramblase-2, FABP (fatty acid-binding protein), IGF-2 (insulin-like growth factor), and MMP-2, and elevating COX-2 at both mRNA and protein expression levels (Cha et al., 2006). Moreover, baicalein notably could append the FGF-21 expression levels via ROR-α which regulates genes related to lipid hemostasis and obesity (Kleiner et al., 2012; Hirai et al., 2019). These findings imply that baicalin is a potent and effective drug to treat obesity and insulin resistance via the Akt/AS160/GLUT4 and P38MAPK/PGC1/GLUT4 pathways (Wang et al., 2021).

**Atherosclerosis**

Atherosclerosis is an inflammatory disease that is associated with an increase in LDL cholesterol levels (Ross, 1999). Atherosclerosis is the number one cause of heart disorders and stroke. In westernized societies, it is responsible for 50% of all deaths (Lusis, 2000). Inflammation and oxidative stress have crucial roles in lipid plaque formation and atherosclerosis (Hajjar and Gotto Jr, 2013). Accordingly, reducing inflammation, oxidative stress, and plasma levels of LDL cholesterol may help prevent and treat atherosclerosis. NLRP3 inflammasome has been suggested to have an important role in the atherosclerosis development. Thus, inhibiting NLRP3 inflammasome may be a potential target to treat atherosclerosis. Zhao et al. did research on the anti-inflammatory and antioxidative stress effects of baicalin and found that baicalin could decrease the expression and activation of NLRP3 inflammasome. They realized that administration of baicalin (50 and 100 mg/kg) could remarkably reduce plaque area. They also found that the treatment with baicalin at doses of 50 and 100 mg/kg could significantly reduce both protein and mRNA levels of NLRP3. In addition, baicalin could remarkably inhibit the production of IL-1, IL-18, total ROS, mitochondria ROS, VCAM-1, and ICAM-1 (Zhao et al., 2020). These findings imply that baicalin inhibits atherosclerosis presumably via the PPAR-LXR-ABCA1/ABCG1 pathway (He et al., 2016).

**Hypertension**

Hypertension is a significant public health concern worldwide, accounting for about 7.5 million deaths, or 12.8% of the world's annual deaths (Singh et al., 2017). The roots of *S. baicalensis* (which has a high amount of baicalein and baicalin) have been reported to lower blood pressure (Lin et al., 1958; Tang and Zhou, 1958). NO-mediated aortic relaxation and cyclic GMP raise are attenuated by baicalin and baicalein, likely via NO-dependent guanylate cyclase activity inhibition (Huang et al., 2004). In a study, baicalin could lower blood pressure in spontaneously hypertensive rats (SHR).* Ex vivo* studies revealed that the endothelium was not required for the vasorelaxant activity of baicalin. Additionally, reduced intracellular levels of Ca^2+^ and increased functionality of K_ATP_ in the vascular smooth muscle under hypertensive conditions might be the mechanisms for the vasorelaxant effect of baicalin (Ding et al., 2019). Moreover, Xue et al. have shown that baicalin may control the TNF-/BMPR2 signaling pathway, providing protection against pulmonary arterial hypertension (Xue et al., 2021).

**Neurological disorders**

**Epilepsy**

Around 50 million people around the world are suffering from epilepsy, which is characterized by unprovoked seizures. Epilepsy is a disease that can affect people of all ages, with a special preference for very young and old people (Sirven, 2015). There is evidence that epilepsy is related to inflammation; epilepsy is often associated with the upregulation of pro-inflammatory genes such as TNF-α, IL-1 and IL-6, and activation of HMGB1, and TLR-4, which are involved in the rapid accumulation of inflammatory mediators (Vezzani et al., 2011; Rana and Musto, 2018; Geis et al., 2019). Baicalin can significantly decrease IL-1 and TNF-α in kainic acid-induced status epilepticus (OuYang et al., 2012). The TLR4/MYD88/Caspase-3 pathway may be activated by baicalein to exert its anti-epileptic actions (Yang et al., 2021).

**Depression**

Depression is a serious behavioral threat to human health. Most patients complain about antidepressant drugs because of their low effects and many adverse effects. Baicalin has exhibited remarkable antidepressant effects in several chronic unpredictable mild stress animal models (Zhang et al., 2016; Gao et al., 2018; Zhang et al., 2018; Lu et al., 2019; Zhong et al., 2019; Jia et al., 2021; Xiao et al., 2021; Fan et al., 2022). In addition, some studies have reported antidepressant effects for baicalein (Table 3).

**Table 3:** Anti-depression properties of baicalin and baicalein in animal models.

Compd.	Model	Result(s)	Mechanism	Dose	Ref.
					
**Baicalin**	CUMS^a^-mice	Improve cognitive functions	↑ p-ERK/ERK and p-CREB/CREB protein ratios ↑ ERK mRNA ↑CREB mRNA expression ↑BDNF/ERK/CREB pathway	25 and 50 mg/kg/d	(Jia et al., 2021)
**Baicalin**	A CUMS-induced mouse model of depression	↓ Depression-like behavior ↑Nerve cells’ survival of the hippocampal dentate gyrus ↑Ki-67- and doublecortin-positive cells	Regulation of the Wnt/β-catenin signaling pathway: ↑ Protein levels of Wingless3a, dishevelled2, and β-catenin ↑ Phosphorylation rate of glycogen synthase kinase-3β and β-catenin nuclear translocation	50 and 100 mg/kg for 21 days, orally	(Xiao et al., 2021)
**Baicalin**	A chronic mild stress (CMS) model of depression	↓Depression-like behavior	Rac/LIMK/cofilin pathway: ↑ PSD95, SYP, TrkB, BDNF, Rac1 and cofilin protein expression levels	50 and 100 mg/kg, intraperitoneal	(Lu et al., 2019)
**Baicalin**	A CUMS-induced mouse model of depression	↓ Depression-like behaviors	↓ Inflammatory cytokines IL1β, IL-6, and TNF-α in serum and hippocampus ↓ NMDAR/NR2B and Ca^2+^/calmodulin-dependent protein kinase II increase ↓ Phosphorylated ERK and ROS decrease	25 and 50 mg/kg	(Zhong et al., 2019)
**Baicalin**	CUMS-induced depression rats	↓ Depressive-like behaviors	↓ Activation of GSK3β/NF-κB/NLRP3 signal pathway: ↑ Doublecortin, neuron-specific enolase, and brain-derived neurotrophic factor levels ↑ Cell survival by reducing MDA level and increasing SOD level ↓ Apoptosis and inflammatory cytokines	_	(Zhang et al., 2018)
**Baicalein**	A lipopolysaccharide-induced mouse model of depression	Attenuate LPS-induced depression-like behavior by suppressing neuroinflammation and inflammation induced by the peripheral immune response	↓ IL-6, TNF-α, MCP-1, and eotaxin production ↓ NF-κB-p65 and iNOS protein levels ↑ Mature brain-derived neurotrophic factor in the hippocampus ↑ mBDNF/proBDNF ratio ↑ CREB expression	A one-time administration (3 mg/kg i.p.)	(Liu et al., 2022)
**Baicalein**	Repeated restraint stress in rats	↓ Duration of immobility in the FST ↑ Sucrose consumption ↑ Dopamine levels	Improve helpless behaviors and depressive symptoms possibly by: ↑ Dopamine and BDNF expression	10, 20, or 40 mg/kg BA (i.p.) 30 min prior to daily exposure to repeated restraint stress (2 h/day) for 14 days	(Lee et al., 2013)

**Ocular diseases**

In the United States, approximately 12 million people aged 40 and up have vision impairment, of which 1 million are blind (Ding et al., 2019). Ocular diseases, for example, cataracts, age-related macular degeneration, DR (diabetic retinopathy), and glaucoma are the main reasons for blindness (Xiao et al., 2014).

**Age-related macular degeneration** **(** **ARMD** **)**

ARMD is a typical chronic, progressive degenerative disorder that destroy central vision of the elderly (Gheorghe et al., 2015). NLRP3 is involved in diverse immune and inflammatory diseases including ARMD. Hong-Jing et al. demonstrated that baicalin has anti-inflammatory and anti-apoptosis effects in Aβ-induced ARPE-19 cells by alleviating pyroptosis through and increase in miR-223 expression and a decrease in NLRP3 expression (Sun et al., 2020).

**Retinopathy of prematurity (ROP)**

ROP is an ocular vascular disease affecting premature infants. The disease is marked by pathological retinal neovascularization, retinal or vitreous haemorrhage, and dilated tortuous retinal blood vessels. It can result in retinal detachment, blindness, and vision impairment (Hartnett and Lane, 2013; Hellström et al., 2013). It has been shown that MMP-2 and MMP-9 play a major role in retinal angiogenesis in retinopathy (Barnett et al., 2007; Rathi et al., 2017). Jo et al. demonstrated that baicalin could essentially inhibit the retinal expression of angiotensin II in a mouse model of oxygen-induced retinopathy. Administration of baicalin (10 mg/kg) could remarkably decrease the expression of MMP-9 and MMP-2, angiotensin II, and VEGF (Jo et al., 2015). They suggested that systemic administration of baicalin can be a potent antiangiogenic compound by inhibiting new vessel formation in the retina. 

**Retinal ischemia**

Retinal ischemia, in its various forms, is a clinical condition and a common cause of visual loss and blindness (Osborne et al., 2004). Baicalein was discovered to exert neuroprotective effect by acting as an antioxidant compound through inhibiting accumulation of ROS in the IAA-induced ischemia model (Maher and Hanneken, 2008). In addition, it might protect against oxidative stress-induced cell death by decreasing the expression level of pro-apoptotic proteins (Ryter et al., 2006; Abraham and Kappas, 2008).

**Diabetic retinopathy (DR)**

The greatest common complication of diabetes mellitus is DR. It is well known for a long time that DR is a microvascular disease (Wang and Lo, 2018). In a DR rodent model, baicalein was able to protect the retina through its anti-inflammatory properties. Baicalein treatment could significantly downregulate the gene expression of TNF-α, IL-18, and IL-1β, decrease the VEGF and GFAP expression in Müller cells, and remarkably decrease vascular abnormalities and ganglion cell loss within the retina (Yang et al., 2009). 

**Neurodegenerative diseases**

Neurodegeneration has been recognized as the main pathophysiological alteration in the majority of brain diseases (Fu et al., 2018). Neurodegenerative illnesses, such as Parkinson's, Alzheimer's, and Huntington's diseases, and amyotrophic lateral sclerosis are neurological problems that presently have no treatment. Protein aggregation, glutamate toxicity, mitochondrial dysfunction, calcium load, oxidative stress, proteolytic stress, aging, and neuroinflammation are all molecular and cellular causes of neuronal death (Muddapu et al., 2020).

**Alzheimer’s disease (AD)**

Baicalein and baicalin have therapeutic effects on behavioral dysfunction of Alzheimer’s disease. They can reduce β-amyloid and trigger non-amyloidogenic amyloid precursor proteins. For instance, administration of baicalin orally for 14 days (100 mg/kg body weight) exhibited neuroprotective effects on pathological changes and behavioral deficits of Aβ 1–42 protein-induced AD in vivo. It could significantly ameliorate memory impairment and attenuate glial cell activation and increase expressions of TNF-α and IL-6, implying its anti-neuroinflammatory activity (Chen et al., 2015).

**Brain ischemia**

Baicalein has a positive effect on alleviating ischemic brain damage. It plays a neuroinflammation role in the pathological process of stroke. Its vital role was unraveled through utilizing different models such as the middle cerebral artery occlusion mouse model and neuroinflammatory microglia model induced by oxygen-glucose deprivation. It can reduce the levels of proinflammatory markers such as CD16, and increase the anti-inflammatory marker CD206 (Ran et al., 2021).

**Parkinson’s disease (PD)**

**Figure 3:**
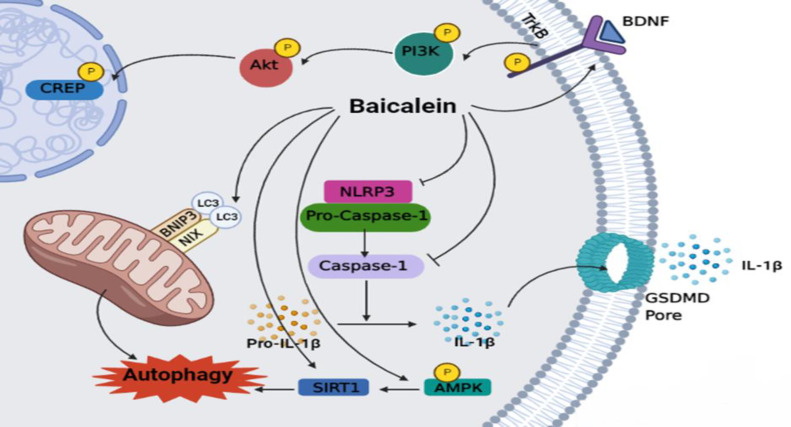
Baicalein attenuates Parkinson’s disease by activating the BDNF/TrkB/CREB pathway, inhibiting NLRP3/Caspase-1/GSDMD Pathway, and induction of mitochondrial autophagy by activating the NIX/BNIP3 pathway, and SIRT1/AMPK/mTOR Pathway.

The neuroprotective effects of baicalein on PD have been investigated in several animal models of study (Table 4). The main underlying mechanism of action for baicalein is illustrated in Figure 3. Activation of the BDNF/TrkB/CREB pathway, inhibition of NLRP3/Caspase-1/GSDMD pathway, and induction of mitochondrial autophagy via activating the NIX/BNIP3 pathway, and SIRT1/AMPK/mTOR pathway are the main mechanisms of action (Rui et al., 2020; Chen et al., 2021; Zhao et al., 2021). 

**Clinical studies**

Despite a vast number of preclinical studies on baicalin and baicalein, very few clinical trials have evaluated the efficacy and safety of these phytochemicals. A randomized, double-blind, placebo-controlled trial (n=374) showed that the administration of baicalin (500 mg/day, orally for 12 weeks) could improve the levels of total cholesterol, TGs, LDLC and apolipoproteins (APOs), and high-sensitivity C-reactive protein (hs-CRP) in patients with rheumatoid arthritis and coronary artery disease (Hang et al., 2018). A single-center, randomized, double-blind, placebo-controlled multiple-ascending-dose study (n=36) has evaluated the tolerability and safety of baicalein tablets in healthy subjects (Li et al., 2021). The subjects received baicalein tablets (200, 400, and 600 mg) or placebo once daily on day 1 and day 10, and 3 times daily on days 4-9. The results of the study showed that administration of baicalein as tablets were generally well-tolerated and safe. 

**Table 4:** The activities of baicalein against Parkinson's disease in animal models.

Model	Mechanism	Dose	Ref.
**MPTP** ^1^ ** induced Parkinson’s disease-like pathology** **Male C57BL/6J mice**	↓ IL-1β, IL-18, IL-6, TNF-α, and iNOS ↓ Caspase-1 activation and NLRP3 Suppress GSDMD	140, 280, or 560 mg/kg, 1 hr before MPTP treatment (9 days) intragastric	(Rui et al., 2020)
**6-Hydroxydopamine induced Parkinson’s disease-like pathology ** **Adult male Sprague-Dawley (SD) rats**	↑ MMP, LC3-II/LC3-I ↓ P62	100 mg/kg every other day (7 days) intraperitoneal	(Chen et al., 2021)
**6-Hydroxydopamine induced Parkinson’s disease -like pathology ** **Adult male SD rats**	↑ LC3-II/LC3-I Reduce P62	100 mg/kg/day (7 days) intraperitoneally	(Chen et al., 2021)
**6-Hydroxydopamine induced Parkinson’s disease -like pathology** ** Adult female SD rats**	↓ Number of rotation speeds and neuron apoptosis ↓ Expression levels of AKT, GSK-3β, α-SYN, and mTOR	25 mg/kg (four weeks) intragastrically	(Zhai et al., 2019)
**Rotenone-induced Parkinson’s disease -like pathology ** **Male C57BL/6J mice**	↓ IFN-γ, IL-1β, IL-4, IL-5, IL-6, IL-10, and IL-12p70 ↑ BDNF/TrkB/CREB pathway	300 mg/kg (4 weeks) orally	(Zhao et al., 2021)

**Pharmacokinetics**

**Absorption**

According to several *in vivo* studies, baicalin is poorly absorbed in the intestine. Because of its glycosidic nature, it cannot pass through the lipid bilayer. When baicalin loses its glucuronide part, its polarity is decreased and consequently, its permeability is increased. Thus, baicalein can be absorbed from the intestinal tract well (Srinivas, 2010; Huang et al., 2019; Ibrahim et al., 2022). Nevertheless, the systemic circulation of baicalin is better than that of baicalein after oral administration. The results from a clinical trial have shown that baicalin levels were increased in circulation after the 

administration of a single dose of baicalein in healthy adults (Li et al., 2014). This is because baicalin and baicalein can transform mutually. After administration, baicalin can be hydrolyzed to baicalein in the intestine by β-glucuronidase. Then, baicalein can be altered to baicalin in the systemic circulation by UDP-glucuronosyltransferase (Zhang et al., 2017). Therefore, the total absorption of baicalin depends on the activity of intestinal bacteria to convert baicalin to baicalein as the first step. However, other factors, including glucuronidation, different absorption sites, and variable gastric emptying might have roles in the absorption of baicalin (Akao et al., 2000; Srinivas, 2010; Huang et al., 2019; Ibrahim et al., 2022). 

**Distribution**

Again, because of its high polarity, baicalin cannot be transported by simple diffusion through the lipid bilayer. Thus, it can be distributed via carriers. According to the literature, probably due to a high protein-binding rate (86–92%), baicalin can be absorbed rapidly into the plasma, but its plasma concentration remains at a certain level (Tang et al., 2006). Baicalein has shown a higher affinity to human serum albumin (HSA), a major transport medium for flavones, than baicalin (Liu et al., 2010). Thus, baicalin can be converted into baicalein to increase its affinity to this carrier. Studies have revealed that multidrug-resistant protein (MRP) 3 and MRP4 can act as basolateral transporters of baicalin, while breast cancer resistance protein (BCRP) and MRP2 might act as the apical transporters of baicalin (Zhang et al., 2007). Kidneys, liver, and lungs are the main organs in which baicalin accumulates the most. The accumulation site for baicalin probably depends on many factors such as routes of administration, preparation, and the multiple herbs present in the preparation. Baicalin and baicalein can pass through the blood brain barrier (BBB) (Zhang et al., 2007). However, due to its high polarity, baicalin should convert into baicalein before passing through the BBB, or it needs active transporters such as organic anion-transporting polypeptide (OATP) 1A2 and OATP2B1 (Srinivas, 2010; Huang et al., 2019; Ibrahim et al., 2022). 

**Metabolism**

The efficacy and toxicity of baicalin depend on its metabolism (Wang et al., 2012; Akao et al., 2013). As mentioned above, β-glucuronidase and UDP-glucuronosyltransferase are the key metabolic enzymes involved in the absorption of baicalin. Sulfatase and catechol-O-methyltransferases are other important metabolic enzymes for the metabolism of baicalin (Wang et al., 2012). Several studies have investigated the metabolism of baicalin (Lu et al., 2012). However, the detection techniques limited these studies. For instance, in a study, baicalin, baicalein, baicalein, 6,7-di-O-β-glucopyranuronoside, and baicalein 6-O-β-d-glucopyranuronoside were detected as the key metabolites after administration of baicalin (Lu et al., 2012). In an in *vivo* model of Zhang et al. investigated and identified the metabolites of baicalin in the serum and plasma of rats (Zhang et al., 2015). In total, 32 metabolites were identified in the serum and plasma samples. Most of the metabolites were distributed in the kidney and liver, showing that baicalin can be metabolized in these organs. Baicalin metabolites were also detected in the heart, spleen, lung, and brain, but at a lower level. In addition, investigating the metabolites revealed that the A ring and 8- and 4′-positions of baicalin are the active metabolic sites (Srinivas, 2010; Zhang et al., 2015; Huang et al., 2019; Ibrahim et al., 2022). Another *in vivo* study has investigated and identified the metabolic metabolites of baicalein after a single dose of the drug by an HPLC-QqTOF technique. In total, seven metabolites including glucuronide, sulfate, glucoside, and methyl-conjugated metabolites were detected, amongst which baicalein-7-O-sulfate and baicalein-6-O-glucuronide-7-O-glucuronide were the most abundant metabolites (Wang et al., 2023). 

**Excretion**

Biliary excretion is the main excretion route of baicalin. Studies have shown that baicalin metabolites are excreted as glucuronides and the process is mediated by multi-drug resistance protein 2 (MRP2) as the main transporter. Renal excretion has also been reported for baicalin metabolites in the form of sulfated and hydroxylated compounds as well as parent drugs (Srinivas, 2010; Huang et al., 2019; Ibrahim et al., 2022). 

## Discussion

The literature review showed that baicalin and baicalein have a wide range of therapeutic activities *in vitro* and *in vivo*. The activities can be generally divided into nine main categories including neurological and neurodegenerative problems, metabolic syndrome, hepatic diseases, cardiovascular problems, infections, cancers, immune system disorders, and ocular diseases. As baicalin and baicalein are flavonoids, most of the activities reported have been attributed to their antioxidant and anti-inflammatory effects. To the best of our knowledge, only two clinical trials have studied the safety and efficacy of these compounds in normal and higher doses (up to 600 mg of oral baicalein). Therefore, further clinical trials are needed to explore the efficacy and safety of these two compounds in different diseases, particularly those mentioned above, on the human population seems necessary. 

*Scutellaria* with 470 accepted species is a cosmopolitan genus. Baicalin is the main component in the roots of these medicinal plants. *Scutellaria baicalensis*, *S. barbata*, and *S. lateriflora* are the most well-known species of the genus., but studies investigating the feasibility of using other *Scutellaria* plants as a source for baicalin and baicalein are limited. For instance, 27 species of the genus (with 11 endemic species) grow widely in Iran, but none of them have been used as a source for baicalin and its homologues. Therefore, conducting further studies on other *Scutellaria* spp. is recommended.

## Conflicts of interest

The authors have declared that there is no conflict of interest.
